# An unexpected player in organ tropism: aspartate functions as signalling molecule to drive lung metastasis

**DOI:** 10.1038/s41392-025-02189-9

**Published:** 2025-03-21

**Authors:** Felix C. E. Vogel, Almut Schulze

**Affiliations:** Division of Tumor Metabolism and Microenvironment, German Cancer Research Center (DKFZ), Heidelberg, Germany

**Keywords:** Lung cancer, Tumour immunology

In a recent study published in *Nature*, Doglioni et al. now reveal an unexpected role for the amino acid aspartate as a signalling molecule that induces alternative translation that ultimately promotes lung metastasis through enhanced collagen secretion.^1^ Metastasis remains the leading cause of cancer-related deaths and many aspects of the metastatic process remain poorly understood.

The metastatic cascade involves several critical steps, including invasion of the cancer cells from the primary tumour into the surrounding tissue, intravasation into the local vasculature, the distribution of disseminated cancer cells by the blood and lymphatic systems, and, finally, their extravasation and outgrowth at a distant site.^2^ The specific targeting of metastases from different cancer types to specific organs, also known as tropism, is governed by numerous factors and has given rise to the *seed-and-soil hypothesis* of metastasis formation, defined over 100 years ago.^3^ Increasing evidence indicates that factors secreted by the primary tumour induce the formation of a pre-metastatic niche within the target organ that promotes the survival and outgrowth of metastatic cells within the target organ.^3^ The pro-metastatic secretome includes growth factors, chemokines and cytokines that promote the remodelling of the extracellular matrix and generate a cancer-permissive immune environment. Moreover, extracellular vesicles (EVs) released by the primary tumour are increasingly recognised as mediators of pre-metastatic niche formation.

The study by Doglioni et al. now demonstrates that the amino acid aspartate is a major mediator of metastasis formation, at least in lung. They initially investigated the mechanism by which tumour-secreted factors (TSF) derived from primary breast cancer tissue slices promote formation of lung metastases using single cell RNA sequencing and found that lung metastatic breast cancer cells from TSF-treated mice express higher levels of genes linked to protein translation. This was associated with elevated levels of lysine-hypusination of eukaryotic initiation factor 5 A (eIF5A). This specific form of post-translational modification is generated by a two-step reaction, using spermidine as a substrate. Interestingly, eIF5A is the only protein for which hypusination has been described. Doglioni et al. demonstrated that silencing of deoxyhypusine synthase (Dhps), the first enzymes mediating eIF5A-hypusination, in murine breast cancer cells efficiently blocks their ability to form lung metastases. The authors then explored the mechanism behind this regulation. Analysis of the chemical composition of lung interstitial fluid showed that TSF-treatment increases aspartate levels. Furthermore, injection of aspartate was sufficient to induced eIF5A hypusination and increase lung metastasis formation in mice. Surprisingly, using tumour-spheroid lines, they found that aspartate was not metabolised, rather the aspartate remains at the cell surface where it functions as a signalling molecule. The proposed receptor involved in aspartate signalling is the N-methyl-D-aspartate (NMDA) receptor, an ionotropic glutamate and glycine receptor usually found in the brain, which induces calcium influx upon activation. One of the two NMDA subunits, Grin2d, was found to be highly expressed in lung metastases and its silencing blocked aspartate-induced eIF5A hypusination and metastasis formation. The authors therefore concluded that aspartate exerts its pro-metastatic function by binding to the NMDA receptor on the surface of metastatic cancer cells.

**Fig. 1:**
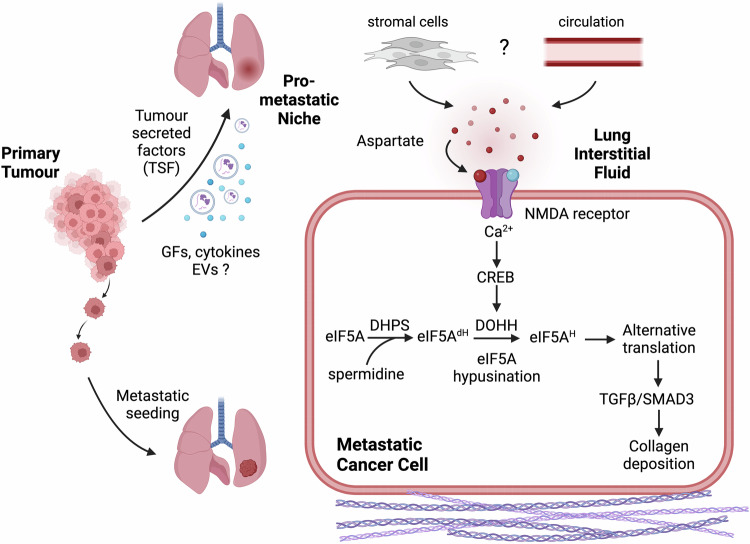
Aspartate functions as extracellular signalling molecule in the pro-metastatic niche of the lung to drive metastasis aggressiveness. Factors secreted by the primary tumour induce the accumulation of aspartate in the interstitial fluid of the lung. Aspartate triggers intracellular calcium signalling by binding to the N-methyl-D-aspartate (NMDA) receptor on the surface of metastatic cancer cells to drive hypusination and activation of the non-classical translation initiation factor eIF5A. Alternative translation induces TGFβ-signalling and induces the synthesis and deposition of collagen fibres. This promotes the formation of a pro-metastatic niche and enhances aggressiveness of lung metastases. GFs growth factors, EVs extracellular vesicles. Figure created with BioRender.com

The authors next dug deeper into unravelling the molecular mechanism downstream of aspartate-induced NMDA receptor signalling. They first showed that the cAMP-responsive element binding protein (CREB) is required for the aspartate-induced expression of deoxyhypusine hydroxylase (Dohh), the second enzyme required for eIF5A hypusination. Furthermore, they demonstrated that aspartate-dependent eIF5A hypusination induces collagen synthesis via activation of transforming growth factor beta (TGFb). Analysis of lung metastases from breast cancer patients provided confirmatory evidence of aspartate signalling in a clinical setting. Together, the reported findings reveal a novel signalling axis involving amino acid signalling, transcription factor activation, protein modification and alternative translation to promote a pro-metastatic state in cancer cells (Fig. 1).

While the study by Doglioni et al. has elucidated the intricate mechanisms by which microenvironmental factors control cancer progression, it also raises further questions. One relates to the exact source of aspartate in the lung microenvironment. The authors observed elevated levels of aspartate in lung interstitial fluid from TSF-treated mice. This may result from growth factors or cytokines secreted by the primary tumour inducing aspartate secretion by stromal cells, or EVs released by the primary tumour. Alternatively, it may be due to reduced uptake and usage by lung tissue cells. Indeed, systemic injection of aspartate phenocopied the effect of TSF-injections and was sufficient to increase aspartate levels in lung interstitial fluid without major effects on circulating aspartate, arguing against a local source. Understanding the source of aspartate is crucial for considering dietary restriction as part of adjuvant treatment for breast cancer lung metastasis. Similarly, the specificity of NMDA receptor activation by aspartate in the lung microenvironment is also not clearly shown. High aspartate levels were detected in interstitial fluids of the liver and bone, but TSF injection did not alter aspartate levels in these tissues. It is unknown if aspartate triggers NMDA receptor activation in these metastatic niches. Use of preclinical models with spontaneous metastasis to liver and/or bone would help to resolve the role of aspartate in these organs. It is possible that microenvironmental factors present in the lung induce Grin2d expression, as this gene was found to be overexpressed in lung compared to other metastatic sites in breast cancer patients. Another question concerns the role of TGFb in promoting metastasis formation downstream of aspartate signalling. TGFb activation is closely linked to epithelial-to-mesenchymal transition (EMT), a process that increases invasion and migration of cancer cells and is central to the metastatic process.^4^ However, EMT is a highly plastic and transient state, and it is generally believed that cancer cells revert to a more epithelial state once they have colonised the target organ. It will therefore be interesting to investigate whether aspartate-induced TGFb activation alters EMT plasticity in cancer cells transiently. Finally, the exact mechanism by which TSF triggers increased aspartate in the lung microenvironment, and not in other organs, remains unclear. Identifying molecules/proteins secreted by cancer cells that increase aspartate in the lung would provide an additional avenue for therapeutic targeting.

Despite these unanswered questions, the results reported in this study have substantial translational potential. The NMDA receptor antagonist memantine is in clinical use to alleviate severe Alzheimer’s disease and has already been found to reduce tumour progression in hepatocellular sarcoma by interfering with the function of tumour-associated macrophages.^5^ Indeed, systemic treatment with memantine resulted in reduced metastasis formation in the experimental system used by Doglioni et al., suggesting that this or related compounds could be investigated clinically. Furthermore, alternative strategies to deplete aspartate levels within the tumour microenvironment could also be explored. This may also be relevant for the clinical use of L-asparaginase that converts asparagine to aspartate and could therefore promote NMDA signalling in certain contexts. In summary, careful investigation is required to elucidate the exact function of non-essential amino acids in cancer.
